# Combined evaluation of Fibrosis‐4 index and fatty liver for stratifying the risk for diabetes mellitus

**DOI:** 10.1111/jdi.13812

**Published:** 2022-05-05

**Authors:** Yasuhiko Todo, Teruki Miyake, Shinya Furukawa, Bunzo Matsuura, Toru Ishihara, Masumi Miyazaki, Akihito Shiomi, Hironobu Nakaguchi, Sayaka Kanzaki, Yasunori Yamamoto, Yohei Koizumi, Osamu Yoshida, Yoshio Tokumoto, Masashi Hirooka, Eiji Takeshita, Teru Kumagi, Yoshio Ikeda, Masanori Abe, Takeru Iwata, Yoichi Hiasa

**Affiliations:** ^1^ Department of Diabetes and Endocrinology Uwajima City Hospital Uwajima Japan; ^2^ Department of Gastroenterology and Metabology Ehime University Graduate School of Medicine Toon Japan; ^3^ Health Services Center Ehime University, Bunkyo Matsuyama Japan; ^4^ Department of Lifestyle‐Related Medicine and Endocrinology Ehime University Graduate School of Medicine Toon Japan; ^5^ Ehime General Health Care Association Matsuyama Japan; ^6^ Postgraduate Medical Education Center Ehime University Graduate School of Medicine Toon Japan

**Keywords:** Diabetes mellitus, Fibrosis, Non‐alcoholic fatty liver disease

## Abstract

**Aims/Introduction:**

To investigate whether the Fibrosis‐4 index can help stratify the risk of diabetes mellitus in patients with fatty liver disease.

**Materials and Methods:**

Based on fatty liver disease and Fibrosis‐4 index (cut‐off value 1.3), we retrospectively divided 9,449 individuals, who underwent at least two annual health checkups, into four groups stratified by sex: normal; high Fibrosis‐4 index without fatty liver disease; low Fibrosis‐4 index with fatty liver disease; and high Fibrosis‐4 index with fatty liver disease.

**Results:**

Onset rates for diabetes mellitus in the normal, high Fibrosis‐4 index without fatty liver disease, low Fibrosis‐4 index with fatty liver disease and high Fibrosis‐4 index with fatty liver disease groups were 1.6%, 4.3%, 6.8% and 10.2%, respectively, in men, and 0.6%, 0.9%, 5.3% and 7.0%, respectively, in women. Compared with the normal group, the high Fibrosis‐4 index without fatty liver disease, low Fibrosis‐4 index with fatty liver disease and high Fibrosis‐4 index with fatty liver disease groups were at a significant risk for diabetes mellitus onset in both male and female participants. Furthermore, in both sexes, high Fibrosis‐4 index with fatty liver disease remained a significant risk factor on multivariate analysis (high fibrosis‐4 index with fatty liver disease group: adjusted hazard ratio 4.03, 95% confidence interval 2.19–7.42 [men] and adjusted hazard ratio 6.40, 95% confidence interval 1.77–23.14 [women]).

**Conclusions:**

Individuals with fatty liver disease and high Fibrosis‐4 index had a higher risk of diabetes mellitus onset. Therefore, Fibrosis‐4 index can help stratify the risk of diabetes mellitus in patients with fatty liver disease and identify patients requiring intervention.

## Graphical abstract

Fatty liver disease is a well‐known risk factor for the development of type 2 diabetes mellitus. However, it has a high morbidity and affects a large number of patients; therefore, it is difficult to provide adequate attention and intervention to all patients. The Fibrosis‐4 index can be used to stratify the risk of developing type 2 diabetes mellitus in patients with fatty liver disease and efficiently identify patients who require intervention.

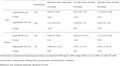

## Introduction

Type 2 diabetes mellitus is considered a risk factor for various diseases, such as diabetic retinopathy, diabetic nephropathy, diabetic neuropathy, atherosclerosis and cancer^1^, ^2^, ^3^, ^4^. Therefore, various efforts have been made to control blood glucose levels and, thereby, inhibit the development of complications. However, the effect of control is not complete, and early intervention is desirable; thus, it is important to appropriately identify patient groups that are at a high risk of developing diabetes mellitus.

Fatty liver disease (FLD) is a well‐known risk factor for the development of type 2 diabetes mellitus^5^, ^6^, ^7^, ^8^. However, it has a high morbidity and affects a large number of patients; therefore, it is difficult to provide adequate attention and intervention to all patients^9^. The Fibrosis‐4 (FIB‐4) index – comprising age, and the levels of platelets, aspartate aminotransferase (AST) and alanine aminotransferase (ALT) – is useful for the assessment of liver fibrosis^10^, ^11^, ^12^. Patients with advanced fibrosis are at high risk of developing hepatocellular carcinoma and liver failure, as well as glucose intolerance^12^, ^13^, ^14^, ^15^, ^16^, ^17^, ^18^. Therefore, the FIB‐4 index – a surrogate marker of fibrosis progression – might be able to stratify the risk of diabetes mellitus in patients with FLD; however, this remains undetermined.

In the present retrospective, longitudinal study, we investigated whether the FIB‐4 index could be used to stratify the risk of developing diabetes mellitus in individuals with FLD.

## Materials and Methods

A total of 9,817 Japanese individuals (4,793 men and 5,024 women) aged 21–78 years were enrolled in this community‐based, longitudinal cohort study. Participants who underwent at least two annual health checks between April 2003 and March 2017 at the Ehime General Health Care Association were eligible for enrollment. During the health check, data pertaining to medical history, medication usage, lifestyle habits, physical measurements and results of routine blood tests were collected. The participants wore a light gown and no footwear when their bodyweight and height measurements were taken. Blood pressure was measured using an automatic sphygmomanometer in the sitting position. Blood samples were collected after fasting for >10 h, and platelet and liver enzymes – such as AST, ALT and gamma‐glutamyl transpeptidase – were analyzed. Lipid profiles were created by measuring triglycerides and high‐density lipoprotein cholesterol. The risk of diabetes mellitus was determined based on the fasting plasma glucose and hemoglobin A1c (HbA1c) levels. The creatinine level was measured to assess renal function.

The FIB‐4 index was calculated using the formula (age × AST [IU/L])/(platelet [10^9^/L] × ALT [IU/L]^1/2^)^10^, ^11^, ^12^. Additionally, health workers asked all participants to complete a questionnaire assessing their medical history; family history of diabetes mellitus (first‐ and second‐degree relatives); medications prescribed; and health‐related behaviors, including exercise habits (no habit or awareness of exercise vs periodic exercise), snacking habits (no snacking vs snacking ≥1 time/day), drinking habits (men ≥210 g/week, women ≥140 g/week) and current smoking habits, before the physical examination^19^. The diagnosis of FLD was made by fourteen experienced technicians using abdominal ultrasonography; the technicians did not have access to the participants' personal data. FLD was diagnosed if there was evidence of two of the four known criteria for the diagnosis of FLD; namely, hepatorenal echocardiographic contrast, liver brightness, deep attenuation and vascular blurring^20^, ^21^.

The present study was approved by the Research Ethics Committee of Ehime University Hospital (#1709007), registered in the Japanese clinical trial registry (UMIN‐CTR; #UMIN000011953) and carried out in accordance with the tenets of the Declaration of Helsinki. The study protocol fulfilled the local ethical and legal requirements, and the Guidelines for Good Clinical Practice. All participant information and data were managed anonymously, and stored in a secure database during the study period. Because this was a retrospective study and all participant data were de‐identified, the ethics committee waived the need to obtain informed consent from the participants.

### Definitions

The FIB‐4 index data were analyzed based on a cut‐off value of 1.3; a lower value indicated a lower risk of having fibrosis; recommendations were made at the first screening for NAFLD^11^, ^12^. Based on the presence of FLD and the cut‐off value for FIB‐4 index using data from the first health checkup, the participants were divided into four groups, by sex: normal (no FLD; FIB‐4 index <1.3); high FIB‐4 without FLD (no FLD; FIB‐4 index ≥1.3); low FIB‐4 with FLD (FLD; FIB‐4 index <1.3); and high FIB‐4 with FLD (FLD; FIB‐4 index ≥1.3).

**Figure 1:**
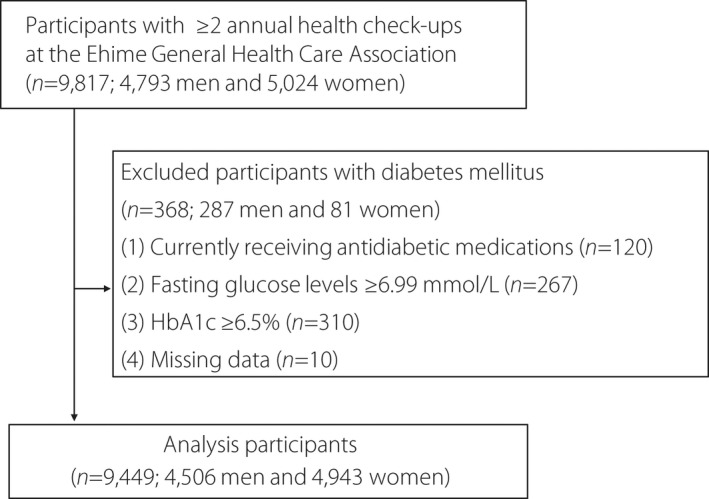
Flowchart for participant selection in this study.

After examining the medical records, 368 individuals with diabetes mellitus who met the following exclusion criteria were excluded: currently receiving antidiabetic medications (*n* = 120), fasting glucose levels ≥6.99 mmol/L (*n* = 267), HbA1c ≥6.5% (*n* = 310),and missing data (*n* = 10; Figure 1). Diabetes mellitus onset was defined as fasting glucose levels ≥6.99 mmol/L, HbA1c ≥6.5% or the start of antidiabetic drug treatment^22^. Finally, the remaining 9,449 individuals (4,506 men and 4,943 women) were analyzed at Ehime University Hospital (Figure 1). The mean duration of observation was 5.53 ± 3.52 years (men 5.32 ± 3.53 years, women 5.73 ± 3.51 years).

JMP software version 14.2.0 (SAS Institute Japan Co. Ltd., Tokyo, Japan) was used for all statistical analyses. Normal assumptions were assessed using the Lilliefors test; however, as no continuous variables were normally distributed, the Steel–Dwass test was used for the analysis; the χ^2^‐test was also used to analyze categorical variables. Univariate Cox proportional hazards regression analyses using the Wald test were carried out to assess the relationship between variables and the development of diabetes mellitus. Multivariate Cox proportional hazards regression analyses, adjusted for body mass index (BMI; kg/m^2^), systolic blood pressure (mmHg), HbA1c (%), creatinine (μmol/L), total cholesterol (mmol/L), triglycerides (mmol/L), exercise habits, snacking habits, drinking habits, smoking status and family history of diabetes mellitus, with or without components of the FIB‐4 index and FLD, were carried out to calculate the adjusted hazard ratios (aHRs) and 95% confidence intervals (CIs) for developing diabetes mellitus. All data are expressed as the median (interquartile range) or number (percentage); a *P*‐value <0.05 was considered to show statistical significance.

## Results

### Baseline characteristics of the participants

Diabetes mellitus onset rates, based on fasting blood sugar (*n* = 96), HbA1c (*n* = 116) and the start of antidiabetic drug treatment (*n* = 27), in the normal, high FIB‐4 without FLD, low FIB‐4 with FLD and high FIB‐4 with FLD groups were 1.6%, 4.3%, 6.8% and 10.2% in men, and 0.6%, 0.9%, 5.3% and 7% in women, respectively (Tables 1 and 2). The high FIB‐4 with FLD group showed the highest incidence of diabetes mellitus (Tables 1 and 2). In both male and female participants, compared with the normal group, the high FIB‐4 without FLD, low FIB‐4 with FLD and high FIB‐4 with FLD groups had significantly older individuals; higher levels of BP, fasting plasma glucose, HbA1c, AST, ALT and total cholesterol; and lower levels of platelets (Tables 1 and 2). In women, triglycerides levels were higher in the high FIB‐4 without FLD, low FIB‐4 with FLD and high FIB‐4 with FLD groups than those in the normal group (Table 2). In men, the proportion of current smokers was higher in the normal group (Table 1).

**Table 1:** Baseline characteristics (men)

	Normal group (*n* = 2,531)	High FIB‐4 index without fatty liver group (*n* = 400)	Low FIB‐4 index with fatty liver group (*n* = 1,418)	High FIB‐4 index with fatty liver group (*n* = 157)	*P*‐value
Age (years)	41 (35–48)	55 (49–58)^†^	43 (37–48.3)^‡,¶^	55 (50–59)^§,††^	<0.01
BMI (kg/m^2^)	22.5 (21–24.1)	22.3 (20.8–24.1)	25.5 (23.8–27.6)^‡,¶^	25.8 (23.8–27.9)^§,††^	<0.01
SBP (mmHg)	114 (105–124)	119 (109–133)^†^	121 (111–133)^‡^	131 (118–144)^§,††,‡‡^	<0.01
DBP (mmHg)	71 (64–80)	78 (69–87)^†^	76 (69–85)^‡^	83 (76.5–93)^§,††,‡‡^	<0.01
FPG (mmol/L)	5.22 (4.94–5.44)	5.33 (5.05–5.66)^†^	5.38 (5.11–5.71)^‡^	5.61 (5.24–5.99)^§,††,‡‡^	<0.01
HbA_1c_ (%)	5.4 (5.2–5.6)	5.5 (5.2–5.7)^†^	5.6 (5.4–5.8)^‡,¶^	5.6 (5.4–5.8)^§,††^	<0.01
Platelets (×10^4^/mm^3^)	23.9 (21.3–27.1)	19.2 (16.5–21.2)^†^	24.7 (21.7–28)^‡,¶^	19.1 (17.3–21.4)^§,‡‡^	<0.01
AST (IU/L)	20 (18–24)	24 (21–29)^†^	24 (20–30)^‡^	32 (25–42.5)^§,††,‡‡^	<0.01
ALT (IU/L)	20 (15–27)	19 (15–25)	33 (24–47)^‡,¶^	34 (22.5–48)^§,††^	<0.01
Cre (μmol/L)	79.6 (70.7–79.6)	79.6 (70.7–88.4)	79.6 (70.7–84.0)	79.6 (70.7–88.4)	
TC (mmol/L)	5.09 (4.55–5.66)	5.22 (4.71–5.82)^†^	5.41 (4.89–5.97)^‡^	5.38 (4.78–5.97)^§^	<0.05
TG (mmol/L)	1.08 (0.78–1.50)	1.06 (0.78–1.52)	1.70 (1.19–2.38)^‡,¶^	1.53 (1.19–2.45)^§,††^	<0.01
Periodic exercise^a^, *n* (%)	925 (36.6%)	188 (47.0%)	403 (28.4%)	58 (36.9%)	<0.01
Snacking habits^b^, *n* (%)	1,098 (43.4%)	136 (34.0%)	705 (49.7%)	57 (36.3%)	<0.01
Drinker^c^, *n* (%)	2,273 (89.8%)	321 (80.3%)	1,298 (91.5%)	108 (68.8%)	<0.01
Current smoker, *n* (%)	1,049 (41.5%)	124 (31.0%)	544 (38.4%)	44 (28.0%)	<0.01
Family history of diabetes, *n* (%)	404 (16.0%)	61 (15.3%)	260 (18.3%)	23 (14.7%)	0.19
Onset of diabetes mellitus^d^, *n* (%)	40 (1.6%)	17 (4.3%)	96 (6.8%)	16 (10.2%)	<0.01

Data are presented as the median (interquartile range) or number (percentage). The Steel–Dwass test was used to analyze continuous variables, and the χ^2^‐test was used to analyze categorical variables. Differences were considered significant at *P* < 0.05 (^†^normal group vs high Fibrosis‐4 [FIB‐4] index without fatty liver group, ^‡^normal group vs low FIB‐4 index with fatty liver group, ^§^normal group vs high FIB‐4 index with fatty liver group, ^¶^high FIB‐4 index without fatty liver group vs low FIB‐4 index with fatty liver group, ^††^high FIB‐4 index without fatty liver group vs high FIB‐4 index with fatty liver group, ^‡‡^low FIB‐4 index with fatty liver group vs high FIB‐4 index with fatty liver group).

^a^ Exercise habit: no habit or awareness of exercise versus periodic exercise.

^b^ Snacking habit: no snacking versus snacking ≥1 time/day.

^c^ Drinkers: men ≥210 g/week, women ≥140 g/week.

^d^ Diabetes mellitus onset was defined as fasting glucose levels ≥6.99 mmol/L, hemoglobin A_1c_ (HbA_1c_) ≥6.5% or the initiation of antidiabetic drug treatment.

ALT, alanine aminotransferase; AST, aspartate aminotransferase; BMI, body mass index; Cre, creatinine; DBP, diastolic blood pressure; FPG, fasting plasma glucose; SBP, systolic blood pressure; TC, total cholesterol; TG, triglycerides.

**Table 2:** Baseline characteristics (women)

	Normal group (*n* = 3,917)	High FIB‐4 index without fatty liver group (*n* = 452)	Low FIB‐4 index with fatty liver group (*n* = 531)	High FIB‐4 index with fatty liver group (*n* = 43)	*P*‐value
Age (years)	39 (34–45)	53 (48–57)^†^	45 (39–51)^‡,¶^	55 (52–58)^§,‡‡^	<0.01
BMI (kg/m^2^)	20.6 (19–22.3)	20.4 (19–21.9)	25.5 (22.8–28.2)^‡,¶^	23.8 (22.1–27.1)^§,††^	<0.01
SBP (mmHg)	105 (97–114)	109 (100–123)^†^	117 (108–131)^‡,¶^	126 (112–141)^§,††^	<0.01
DBP (mmHg)	64 (58–71.5)	68 (60–76)^†^	73 (65–81)^‡,¶^	79 (69–85)^§,††^	<0.01
FPG (mmol/L)	4.88 (4.61–5.11)	4.94 (4.72–5.22)^†^	5.22 (4.94–5.55)^‡,¶^	5.22 (4.88–5.92)^§,††^	<0.01
HbA_1c_ (%)	5.4 (5.2–5.6)	5.5 (5.2–5.7)^†^	5.7 (5.5–5.9)^‡,¶^	5.7 (5.5–5.9)^§,††^	<0.01
Platelets (×10^4^/mm^3^)	25 (21.7–28.6)	19.4 (17.3–21.7)^†^	27.4 (24.1–32.2)^‡,¶^	19.1 (16.7–22)^§,‡‡^	<0.01
AST (IU/L)	18 (16–20)	22 (20–26)^†^	20 (17–24)^‡,¶^	25 (22–31)^§,††,‡‡^	<0.01
ALT (IU/L)	13 (11–17)	16 (13–20)^†^	20 (15–28)^‡,¶^	24 (17–38)^§,††^	<0.01
Cre (μmol/L)	53.0 (53.0–61.9)	60.1 (53.0–61.9)^†^	53.0 (53.0–61.9)^¶^	59.2 (53.0–61.9)	<0.01
TC (mmol/L)	5.07 (4.53–5.66)	5.61 (5.02–6.23)^†^	5.48 (4.97–6.10)^‡^	5.66 (5.30–6.34)^§^	<0.01
TG (mmol/L)	0.70 (0.54–0.94)	0.80 (0.60–1.08)^†^	1.18 (0.78–1.71)^‡,¶^	1.13 (0.98–1.48)^§,††^	<0.01
Periodic exercise^a^, *n* (%)	1,004 (25.6%)	186 (41.2%)	121 (22.8%)	11 (25.6%)	<0.01
Snacking habits^b^, *n* (%)	3,344 (85.4%)	378 (83.6%)	461 (86.8%)	34 (79.1%)	0.34
Drinker^c^, *n* (%)	189 (4.8%)	28 (6.2%)	27 (5.1%)	1 (2.3%)	0.52
Current smoker, *n* (%)	218 (5.6%)	21 (4.7%)	38 (7.2%)	0 (0%)	0.12
Family history of diabetes, *n* (%)	862 (22.0%)	74 (16.4%)	150 (28.3%)	12 (27.9%)	<0.01
Onset of diabetes mellitus^d^, *n* (%)	24 (0.6%)	4 (0.9%)	28 (5.3%)	3 (7.0%)	<0.01

Data are presented as the median (interquartile range) or number (percentage). The Steel–Dwass test was used to analyze continuous variables, and the χ^2^‐test was used to analyze categorical variables. Differences were considered significant at *P* < 0.05 (^†^normal group vs high Fibrosis‐4 [FIB‐4] index without fatty liver group, ^‡^normal group vs low FIB‐4 index with fatty liver group, ^§^normal group vs high FIB‐4 index with fatty liver group, ^¶^high FIB‐4 index without fatty liver group vs low FIB‐4 index with fatty liver group, ^††^high FIB‐4 index without fatty liver group vs high FIB‐4 index with fatty liver group, ^‡‡^low FIB‐4 index with fatty liver group vs high FIB‐4 index with fatty liver group).

^a^ Exercise habit: no habit or awareness of exercise versus periodic exercise.

^b^ Snacking habit: no snacking versus snacking ≥1 time/day.

^c^ Drinkers: men ≥210 g/week, women ≥140 g/week.

^d^ Diabetes mellitus onset was defined as fasting glucose levels ≥6.99 mmol/L, hemoglobin A_1c_ (HbA_1c_) ≥6.5% or the initiation of antidiabetic drug treatment.

ALT, alanine aminotransferase; AST, aspartate aminotransferase; BMI, body mass index; Cre, creatinine; DBP, diastolic blood pressure; FPG, fasting plasma glucose; SBP, systolic blood pressure; TC, total cholesterol; TG, triglycerides.

### Risk of the FIB‐4 index components and FLD for diabetes mellitus onset

Univariate analysis showed that age, AST, ALT, high FIB‐4 index and FLD were significant risk factors for diabetes mellitus (age, hazard ratio [HR] 1.07, 95% CI 1.05–1.09; AST, HR 1.011, 95% CI 1.008–1.014; ALT, HR 1.011, 95% CI 1.009–1.013; FIB‐4 index, HR 2.78, 95% CI 1.89–4.07; and FLD, HR 4.18, 95% CI 3.03–5.75) in men (Table 3). Further, age, platelets, AST, ALT and FLD were significant risk factors for diabetes mellitus (age, HR 1.10, 95% CI 1.07–1.13; platelets, HR 1.07, 95% CI 1.03–1.10; AST, HR 1.02, 95% CI 1.001–1.03; ALT, HR 1.015, 95% CI 1.006–1.021; and FLD, HR 10.3, 95% CI 6.18–17.20) in women (Table 4). Additionally, age and FIB‐4 index in men, and fatty liver in women remained significant risk factors on multivariate analysis (age, adjusted HR 1.05, 95% CI 1.03–1.07; FIB‐4 index, adjusted HR 2.98, 95% CI 1.97–4.52 [Table 3] and FLD, adjusted HR 2.51, 95% CI 1.30–4.84 [Table 4]).

**Table 3:** Risk of the Fibrosis‐4 index components and fatty liver disease for diabetes mellitus onset (men)

	Crude HR (95% CI)	*P*‐value	Adjusted HR^†^ (95% CI)	*P*‐value
Age (years)	1.07 (1.05–1.09)	<0.01	1.05 (1.03–1.07)	<0.01^‡^
Platelets (×10^4^/mm^3^)	1.01 (0.98–1.04)	0.73	0.99 (0.96–1.02)	0.38^§^
AST (IU/L)	1.011 (1.008–1.014)	<0.01	1.00 (0.99–1.02)	0.47^¶^
ALT (IU/L)	1.011 (1.009–1.013)	<0.01	1.007 (0.999–1.015)	0.11^††^
FIB‐4 index (≥1.3)	2.78 (1.89–4.07)	<0.01	2.98 (1.97–4.52)	<0.01^‡‡^
Fatty liver	4.18 (3.03–5.75)	< 0.01	1.23 (0.86–1.75)	0.26^§§^

Differences were considered statistically significant at *P* < 0.05.

^†^ Multivariate Cox proportional hazards regression analysis adjusted for body mass index (kg/m^2^), systolic blood pressure (mmHg), hemoglobin A_1c_ (%), creatinine (μmol/L), total cholesterol (mmol/L), triglycerides (mmol/L), exercise habits, snacking habits, drinking habits, smoking habits and family history of diabetes, along with either:

^‡^ Platelets, aspartate aminotransferase (AST), alanine aminotransferase (ALT) and fatty liver disease.

^§^ Age, AST, ALT and fatty liver disease.

^¶^ Age, platelet, ALT and fatty liver disease.

^††^ Age, platelet, AST and fatty liver disease.

^‡‡^ Fatty liver disease.

^§§^ Age, platelet count, AST and ALT.

CI, confidence interval; Cre, creatinine; FIB‐4, Fibrosis‐4; HR, hazard ratio.

**Table 4:** Risk of the Fibrosis‐4 index components and fatty liver disease for diabetes mellitus onset (women)

	Crude HR (95% CI)	*P*‐value	Adjusted HR^†^ (95% CI)	*P*‐value
Age (years)	1.10 (1.07–1.13)	<0.01	1.05 (1.03–1.07)	0.07^‡^
Platelet (×10^4^/mm^3^)	1.07 (1.03–1.10)	<0.01	0.99 (0.97–1.01)	0.48^§^
AST (IU/L)	1.02 (1.001–1.03)	<0.01	1.003 (0.989–1.016)	0.22^¶^
ALT (IU/L)	1.015 (1.006–1.021)	<0.01	1.007 (0.998–1.015)	0.36^††^
FIB‐4 index (≥1.3)	1.69 (0.77–3.73)	0.19	1.43 (0.61–3.38)	0.41^‡‡^
Fatty liver	10.3 (6.18–17.20)	<0.01	2.51 (1.30–4.84)	<0.01^§§^

Differences were considered statistically significant at *P* < 0.05.

^†^ Multivariate Cox proportional hazards regression analysis adjusted for body mass index (kg/m^2^), systolic blood pressure (mmHg), hemoglobin A_1c_ (%), creatinine (μmol/L), total cholesterol (mmol/L), triglycerides (mmol/L), exercise habits, snacking habits, drinking habits, smoking habits and family history of diabetes, along with either:

^‡^ Platelet, aspartate aminotransferase; (AST), alanine aminotransferase (ALT) and fatty liver disease.

^§^ Age, AST, ALT and fatty liver disease.

^¶^ Age, platelet, ALT and fatty liver disease.

^††^ Age, platelet, AST and fatty liver disease.

^‡‡^ Fatty liver disease.

^§§^ Age, platelet count, AST and ALT.

CI, confidence interval; Cre, creatinine; FIB‐4, Fibrosis‐4; HR, hazard ratio.

### Risk of diabetes mellitus onset in participants with FLD and a high FIB‐4 index

Compared with the participants in the normal group, those in the high FIB‐4 without FLD, low FIB‐4 with FLD and high FIB‐4 with FLD groups were at a significant risk for diabetes mellitus onset (high FIB‐4 without FLD group, HR 4.54, 95% CI 2.57–8.03; low FIB‐4 with FLD group, HR 5.07, 95% CI 3.50–7.33; and high FIB‐4 with FLD group, HR 11.73, 95% CI 6.56–21.00 in men [Table 5]; high FIB‐4 without FLD group, HR 2.08, 95% CI 0.72–6.00; low FIB‐4 with FLD group, HR 10.70, 95% CI 6.19–18.47; and high FIB‐4 with FLD group, HR 17.37, 95% CI 5.22–57.85 in women [Table 5]). Additionally, on multivariate analysis adjusted for BMI, systolic blood pressure, HbA1c, creatinine, exercise habit, snacking habit, drinking habit, smoking habit and family history of diabetes, these relationships remained significant in men in the high FIB‐4 without FLD and high FIB‐4 with FLD groups, and in women in the low FIB‐4 with FLD and high FIB‐4 with FLD groups (men high FIB‐4 without FLD group, aHR 3.27, 95% CI 1.80–5.95; high FIB‐4 with FLD group, aHR 4.03, 95% CI 2.19–7.42 [Table 5]; women low FIB‐4 with FLD group, aHR 2.26, 95% CI 1.12–4.57; high FIB‐4 with FLD group, aHR 6.40, 95% CI 1.77–23.14, [Table 5]).

**Table 5:** Risk of diabetes mellitus onset in patients with fatty liver disease and a high Fibrosis‐4 index

	Normal group	High FIB‐4 index without fatty liver group	Low FIB‐4 index with fatty liver group	High FIB‐4 index with fatty liver group
Men				
Crude HR (95% CI) *P*‐value	1.00	4.54 (2.57–8.03) <0.01	5.07 (3.50–7.33) <0.01	11.73 (6.56–21.00) <0.01
Adjusted HR^†^ (95% CI) *P*‐value	1.00	3.27 (1.80–5.95) <0.01	1.45 (0.97–2.17) 0.07	4.03 (2.19–7.42) <0.01
Women				
Crude HR (95% CI) *P*‐value	1.00	2.08 (0.72–6.00) 0.18	10.70 (6.19–18.47) <0.01	17.37 (5.22–57.85) <0.01
Adjusted HR^†^ (95% CI) *P*‐value	1.00	1.006 (0.33–3.03) 0.99	2.26 (1.12–4.57) 0.02	6.40 (1.77–23.14) <0.01

Differences were considered statistically significant at *P* < 0.05.

^†^ Multivariate Cox proportional hazards regression analysis was adjusted for body mass index (kg/m^2^), systolic blood pressure (mmHg), hemoglobin A_1c_ (%), creatinine (μmol/L), total cholesterol (mmol/L), triglycerides (mmol/L), exercise habits, snacking habits, drinking habits, smoking habits and family history of diabetes.

CI, confidence interval; FIB‐4, Fibrosis‐4; HR, hazard ratio.

In the normal, high FIB‐4 without FLD, low FIB‐4 with FLD and high FIB‐4 with FLD groups, diabetes mellitus onset rates were 0.20%, 0.79%, 0.37% and 0% at 1 year; 0.53%, 3.84%, 2.28% and 7.67% at 3 years; 1.12%, 5.85%, 4.32% and 13.92% at 5 years; 3.07%, 8.94%, 24.43% and 43.12% at 10 years; and 4.81%, 8.94%, 15.79% and 24.17% at 12 years, respectively, in men. The diabetes mellitus onset rates in women in the normal, high FIB‐4 without FLD, low FIB‐4 with FLD and high FIB‐4 with FLD groups were 0.03%, 0%, 0.78% and 0% at 1‐year; 0.09%, 0%, 2.70% and 5.95% at 3 years; 0.48%, 0.92%, 4.05% and 5.95% at 5 years; and 1.32%, 1.64%, 9.79% and 5.95% at 10 years; 1.66%, 4.37%, 15.70% and 24.76% at 12 years, respectively, in women.

## Discussion

The present study examined the combined effect of FLD and FIB‐4 index on diabetes mellitus onset by sex, and showed that individuals with FLD with a high FIB‐4 index had a higher risk for diabetes mellitus onset than those without FLD with a low FIB‐4 index, those without FLD with a high FIB‐4 index, and those with FLD with a low FIB‐4 index among men and women; therefore, the FIB‐4 index could be used to stratify the risk of diabetes mellitus in individuals with FLD, and the combination of FIB‐4 index and fatty liver can help identify individuals at a high risk for developing diabetes mellitus.

The FIB‐4 index is widely used as a simple predictive marker of fibrosis in the liver, and is associated with liver‐related diseases and pathophysiology of diabetes mellitus. In a longitudinal study, Ampuero *et al*.^23^ enrolled 178 metabolically healthy patients diagnosed with biopsy‐proven non‐alcoholic fatty liver disease (without type 2 diabetes mellitus, arterial hypertension or dyslipidemia at the baseline), and examined its effect on the development of type 2 diabetes mellitus, arterial hypertension and dyslipidemia. Although metabolically healthy non‐alcoholic fatty liver disease patients with significant fibrosis showed a higher risk for type 2 diabetes mellitus onset and arterial hypertension, FIB‐4 index could not predict the occurrence of type 2 diabetes mellitus^23^. However, it is possible that no significant association was found due to the small sample size of the study. Conversely, Sung *et al*.^24^ followed up 70,303 patients without obesity (BMI <25 kg/m^2^) or diabetes mellitus, and examined the effects of FLD, insulin resistance (homeostatic model assessment [HOMA]‐IR ≥2.0), central obesity (waist circumference men ≥90 cm, women ≥85 cm) and liver fibrosis (FIB‐4 index) on the development of diabetes mellitus. They observed that the combination of FLD, insulin resistance and the highest quartile of FIB‐4 index significantly increased the risk of diabetes mellitus onset in men, but not in women^24^.

The mechanism by which the FIB‐4 index enhances the risk of diabetes mellitus onset in individuals with FLD might be associated with its effect on insulin secretion. Fujita *et al*.^17^ recruited 1,268 participants without diabetes mellitus for a cross‐sectional study and 439 participants for a longitudinal study, and examined the association between FIB‐4 index and glucose metabolism. In the cross‐sectional study, FIB‐4 index correlated with HOMA‐β and HOMA‐R, whereas in the longitudinal study, a high FIB‐4 index (≥1.592) was associated with a decrease in insulin (HOMA‐β <30)^17^.

In a previous study, our colleagues examined 20 patients with and 21 patients without cirrhosis; they compared them with delta C‐peptide immunoreactivity using the glucagon challenge test and pancreatic perfusion parameters through dynamic contrast‐enhanced ultrasound^18^. Additionally, we previously investigated the islet insulin secretion and thickness of the pancreatic vein using the autopsy specimens of 20 and 23 individuals with and without cirrhosis, respectively^18^. Our results showed that portal hypertension in patients with cirrhosis was associated with the pancreatic drainage time measured using dynamic contrast‐enhanced ultrasound. A longer pancreatic drainage time showed a negative correlation with delta C‐peptide immunoreactivity. Histopathological examination showed that insulin‐positive areas in islets decreased in patients with cirrhosis compared with those in patients without cirrhosis; in addition, the number of insulin‐positive cells in the pancreas was negatively associated with the pancreatic venous thickness^18^. These results suggest that the progression of fibrosis in the liver induces pancreatic congestion, decreases insulin secretion and induces glucose intolerance.

The present study had several strengths; first, it was a community‐based study. Second, health workers asked all participants to complete a questionnaire before physical examination; thus, although this was a large study, there were limited missing data (BMI value [*n* = 1], platelet values [*n* = 5], data regarding exercise habits [*n* = 2] and data regarding snacking habits [*n* = 2]). However, the present study had several limitations. First, although abdominal ultrasonography can diagnose FLD with a high sensitivity and specificity^25^, ^26^, we did not have data regarding fatty deposition in the liver; thus, we could not assess the relationship between the severity of FLD and diabetes mellitus onset. Second, this survey used self‐reported data for several factors, which might have compromised the accuracy of the survey results. Third, the data were not truly continuous, as they were only collected once a year during the annual health checkup. Fourth, FIB‐4 index is sensitive to age. Accordingly, its diagnostic performance might not be the same for young individuals. Fifth, our data did not include the waist circumference, and could not assess other easy and useful surrogate markers of fatty liver, such as the Fatty Liver Index. Sixth, our data did not include information about nutritional status, accumulated alcohol consumption and types of alcoholic beverages. Finally, as the entire study population was Japanese, studies including participants of other racial backgrounds should be carried out to validate the present findings.

In conclusion, the risk of diabetes mellitus onset is higher for both men and women with FLD and a high FIB‐4 index than in those with a high FIB‐4 index, but no FLD, as well as those with FLD and a low FIB‐4 index. Thus, the FIB‐4 index can be used to stratify the risk of developing diabetes mellitus in patients with FLD and efficiently identify patients who require intervention.

## Disclosure

The authors declare no conflict of interest.

Approval of the research protocol: This study was approved by the Research Ethics Committee of Ehime University Hospital (approval number: 1709007), and it conforms to the provisions of the Declaration of Helsinki.

Informed consent: N/A. As this study protocol was retrospective in manner and all participants’ data were de‐identified, it was not necessary to obtain informed consent from participants in this study.

Registry and the registration no. of the study/trial: 3 October 2013. University Hospital Medical Information Network ID: UMIN000011953.

Animal studies: N/A.
